# Platypnea-Orthodeoxia Syndrome Caused by a Patent Foramen Ovale and Interatrial Septal Aneurysm: A Case Report

**DOI:** 10.7759/cureus.112903

**Published:** 2026-07-18

**Authors:** Abes A Bautista Neughebauer, Raymond L Benza, Deepak Talreja

**Affiliations:** 1 Internal Medicine, Eastern Virginia Medical School, Norfolk, USA; 2 Cardiology, Eastern Virginia Medical School, Norfolk, USA

**Keywords:** amplatzer, interatrial aneurysm, orthodeoxia, pfo, platypnea

## Abstract

Platypnea-orthodeoxia syndrome (POS) is a rare condition characterized by positional hypoxemia, often due to right-to-left shunting across a patent foramen ovale (PFO) despite normal right-sided pressures. Early recognition is essential as symptoms may be subtle and imaging is often unrevealing. A 78-year-old female patient with a known PFO presented with one week of lower abdominal pain and mild exertional dyspnea without chest pain. She was profoundly hypoxic to 76% on room air, with improvement when supine and worsening when upright. Initial labs were unremarkable, and arterial blood gas revealed primary respiratory alkalosis with severe hypoxemia. Despite unremarkable chest radiography, CT angiography, and ECG, oxygen via high-flow nasal cannula at 100% FiO_2_ only minimally improved her SpO_2_, prompting consideration of right-to-left shunt physiology. Prior transthoracic echocardiograms had documented a small PFO without evidence of pulmonary hypertension or right-sided disease. Repeat echocardiogram demonstrated an atrial septal aneurysm and a positive bubble study. Transcatheter PFO closure was performed, and oxygen saturation improved immediately. She was weaned from the nasal cannula over three days. Post-procedural echocardiogram showed no residual shunt. This case highlights the importance of recognizing POS as a potential presentation of PFO-related right-to-left shunting, particularly when hypoxemia is refractory to supplemental oxygen and standard cardiopulmonary evaluation is unrevealing. Early closure can lead to dramatic clinical improvement.

## Introduction

Platypnea-orthodeoxia syndrome (POS) is a rare clinical entity characterized by dyspnea and arterial desaturation that worsen in the upright position and improve when supine [1,2]. Positional hypoxemia occurs because upright posture can alter intracardiac geometry and venous flow, redirecting deoxygenated blood across a right-to-left shunt (RLS) and bypassing pulmonary oxygenation. Early recognition is clinically important because delayed diagnosis may lead to prolonged, unnecessary pulmonary evaluation and postponement of definitive therapy. Although hypoxemia is commonly attributed to pulmonary pathology, POS should prompt consideration of right-to-left shunting, particularly when oxygen saturation fails to correct with supplemental oxygen. A key diagnostic clue is hypoxemia that is refractory to supplemental oxygen and demonstrates positional variation, underscoring the importance of measuring oxygen saturation in both supine and upright positions. Intracardiac causes most often involve interatrial communications such as atrial septal defects (ASDs) or patent foramen ovale (PFO), although additional anatomic or functional factors are usually required to redirect venous blood across the defect [1-3].

PFO is common in the general population but rarely causes clinically significant hypoxemia in the absence of elevated right-sided pressures. However, distortion of atrial anatomy from conditions such as atrial septal aneurysm (ASA), aortic root or ascending aortic dilation, kyphoscoliosis, or prominent Eustachian valve/Chiari network may facilitate positional right-to-left shunting [1,2]. We present a case of severe refractory positional hypoxemia due to POS in a patient with known PFO, ASA, and proximal ascending aortic dilation, successfully treated with transcatheter PFO closure.

## Case presentation

A 78-year-old female patient with a past medical history significant for diverticulosis, hypertension, hyperlipidemia, hypothyroidism status post thyroidectomy on levothyroxine, and a known small PFO since 2013, presented with one week of lower abdominal pain. In the emergency department, she was found to be hypoxic at 76% while on room air (RA) and endorsed mild shortness of breath on exertion but denied chest pain. Her vital signs on presentation included heart rate 104 beats per minute, respiratory rate 17 respirations per minute, blood pressure 141/91 mmHg, and oxygen saturation (SpO_2_) of 76% on RA while upright. Interestingly, it was observed by the care team that her SpO_2_ improved to 91% while supine early in the admission but dropped when she sat upright. On appearance, she was visibly cyanotic in the upper and lower extremity digits. She was able to speak easily in full sentences. On auscultation, she was found to be tachycardic with a regular rhythm without murmurs. Her lungs were clear to auscultation bilaterally, and her abdomen was nontender and without evidence of pitting edema.

Basic laboratory work was pursued. Her complete blood count and basic metabolic panel were within normal limits, without any findings of leukocytosis, anemia, elevated creatinine, or electrolyte derangements. The first serum troponin measurement was 20 ng/L, and repeat measurements were 19 ng/L and 19 ng/L, all within normal limits. Her serum N-terminal pro-B-type natriuretic peptide was 180 pg/mL, within normal limits for her age group. An arterial blood gas (ABG) was obtained and was notable for primary respiratory alkalosis with compensatory metabolic acidosis and specifically pH of 7.480, PCO_2_ 23.7 mmHg, PaO_2_ 45 mmHg, bicarbonate 17.6 mEq/L (Table 1). The patient’s chest X-ray was remarkable only for low lung volumes and negative for cardiomegaly or pulmonary edema (Figure 1a). Her electrocardiogram (ECG) did not demonstrate any signs of ischemia or arrhythmia (Figure 1b). A CT angiography was then obtained, which was not notable for any lung pathology or pulmonary embolism (Figure 2). Interestingly, she had recurrent visits to the emergency department for dizziness and mild dyspnea with exertion, with the most recent episode being four months prior. At that time, complete pulmonary workup, including ABG, chest X-ray, and CT chest, was negative.

**Table 1:** Laboratory results. NT-proBNP: N-terminal pro-B-type natriuretic peptide

Complete Blood Count	Result	Reference Range	Units
White Blood Cell count	6.3	4.0-11.0	×10³/µL
Hemoglobin	16.2	11.7-16.1	g/dL
Hematocrit	47.7	35.1-48.3	%
Mean Corpuscular Volume	98	80-99	fL
Platelets	204	140-440	×10³/µL
Basic Metabolic Panel			
Glucose	126	70-99	mg/dL
Blood Urea Nitrogen	12	6-22	mg/dL
Creatinine	1.1	0.8-1.4	mg/dL
Sodium	134	133-145	mmol/L
Potassium	3.7	3.5-5.5	mmol/L
Chloride	97	98-110	mmol/L
Bicarbonate	18	20-32	mmol/L
Calcium	9.7	8.4-10.5	mg/dL
Anion Gap	19	3-15	mmol/L
Liver Function Panel			
Aspartate Aminotransferase (AST)	22	10-37	U/L
Alanine Aminotransferase (ALT)	8	5-40	U/L
Alkaline Phosphatase	103	40-120	U/L
Total Bilirubin	0.6	0.2-1.2	mg/dL
Direct Bilirubin	0.2	0-0.3	mg/dL
Total Protein	7.4	6.2-8.1	g/dL
Albumin	4.4	3.5-5.0	g/dL
Lipase	<20	7-60	U/L
Cardiac Markers			
NT-proBNP	180	≤450	pg/mL
High-Sensitivity Troponin Trend	20 → 19 → 19	<20	ng/L
Arterial Blood Gas			
pH	7.480	7.35-7.45	-
PaCO₂	23.7	34-45	mmHg
PaO₂	45	80-100	mmHg
HCO₃⁻	17.6	22-26	mmol/L
Base Excess	-6.0	-2 to +2	mmol/L

**Figure 1:**
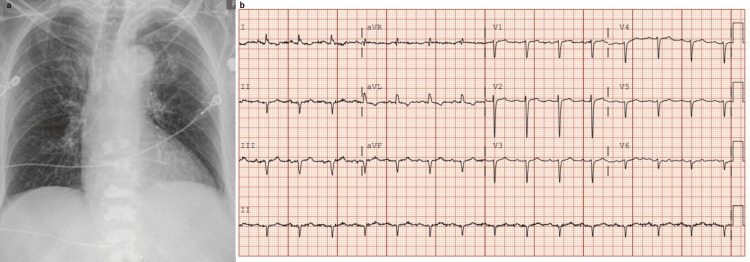
(a) Chest radiograph obtained on admission demonstrating low lung volumes without cardiomegaly or significant pulmonary abnormalities. (b) Electrocardiogram obtained on admission showing normal sinus rhythm with no ST-T segment changes or arrhythmias.

**Figure 2:**
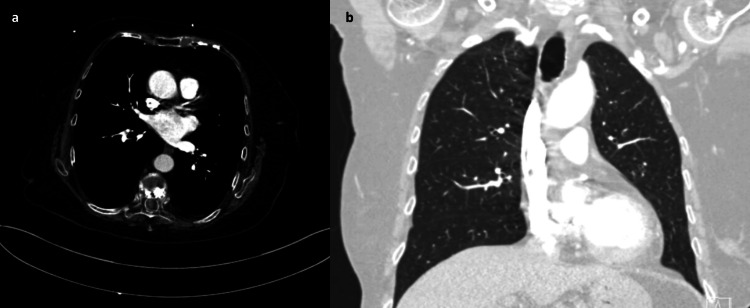
Admission computed tomography angiography (CTA) demonstrating (a) no evidence of pulmonary embolism and (b) no significant pulmonary pathology.

She was placed on high-flow nasal cannula (HFNC) at 100% inspired fraction of oxygen (FiO_2_), and her saturation improved only minimally from 79% on RA to 82% on HFNC, without any change in clinical symptoms. She denied a history of tobacco use, but recalled being discharged on oxygen a year prior for unknown reasons and had discontinued it herself as she was feeling well. Alternative causes of hypoxemia were considered but were felt to be less likely based on the available evaluation and workup. Pulmonary embolism and major parenchymal lung disease were excluded by CT angiography, chest radiography showed no pulmonary edema or cardiomegaly, and cardiac biomarkers were not suggestive of acute coronary syndrome or decompensated heart failure. Further workup with serum methemoglobin and carboxyhemoglobin testing was unrevealing. The combination of marked positional desaturation and minimal improvement despite 100% FiO_2_ suggested a shunt physiology and underlying cardiac cause, prompting further workup. Given that there was no significant improvement in oxygen saturation with supplemental oxygen, a cardiac etiology with a likely shunt was entertained as the most likely diagnosis.

Her past transthoracic echocardiograms (TTE) were significant for PFO but did not demonstrate any right atrial/ventricular dilation or pulmonary hypertension to make an RLS very likely on the differential (Figure 3a). Follow-up TTE showed an ejection fraction (EF) of 70%, a positive bubble study, undetermined pulmonary artery systolic pressure (because of inadequate tricuspid regurgitation), an ASA (Figure 3b), and a dilated proximal ascending aorta (3.4 cm with an index of 1.8 cm/m^2^).

**Figure 3:**
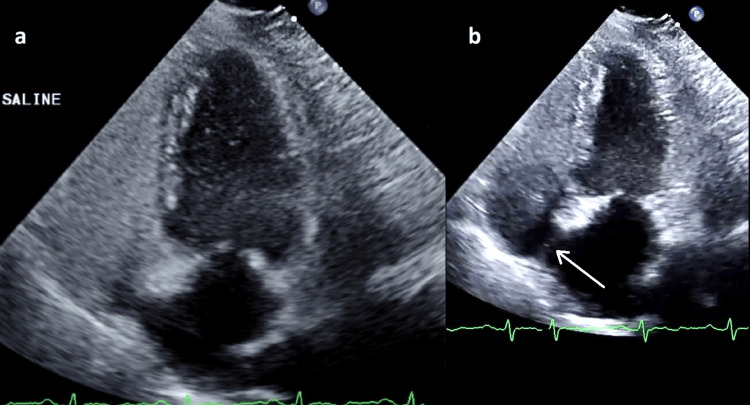
(a) Echocardiogram prior to presentation showing a positive saline bubble study indicative of patent foramen ovale (PFO); (b) admission echocardiogram showing the interatrial septal aneurysm (arrow).

At this point, intracardiac shunting was the most logical culprit, and the decision was made to close her PFO via a transcatheter approach. An Amplatzer 30 mm PFO Occluder device (Abbott Cardiovascular, IL, USA) was used. After sedation, a standard 8-French (8F) right femoral venous sheath and an 8F long RF venous sheath were placed. Fluoroscopy was used to analyze the PFO, which was found to be 10 mm in size. The defect was crossed with a multipurpose guide catheter and an angled Glidewire guidewire (Terumo Medical Corporation, Somerset, NJ, USA). Balloon sizing was performed, and an Amplatzer 30 mm device was advanced across the atrial septum over an Amplatz stiff wire. The left atrial side was first deployed, followed by the right atrial side. The deployment is seen in Figure 4a. She had an immediate improvement in her SpO_2_ to 100% while in the cath lab. There was no shunting seen on intracardiac echocardiography (ICE) (Figure 4b). She was placed on HFNC with 40% FiO_2_, and eventually weaned to 2 liters nasal cannula over the next three days (Table 2). Repeat TTE was obtained the next day, showing no residual shunt. The reported abdominal pain was not associated with tenderness on physical examination, leukocytosis, or concerning imaging findings, and it resolved upon further clinical improvement. A six-minute walk test showed 93% oxygen saturation at rest and 91% while ambulating. She was discharged with dual antiplatelet therapy (DAPT) for six months, followed by aspirin monotherapy indefinitely. DAPT was selected to reduce the risk of device-associated thrombus during the early post-implantation period, when endothelialization and epithelialization of the occluder are incomplete. After this initial healing phase, therapy was planned to transition to aspirin monotherapy.

**Figure 4:**
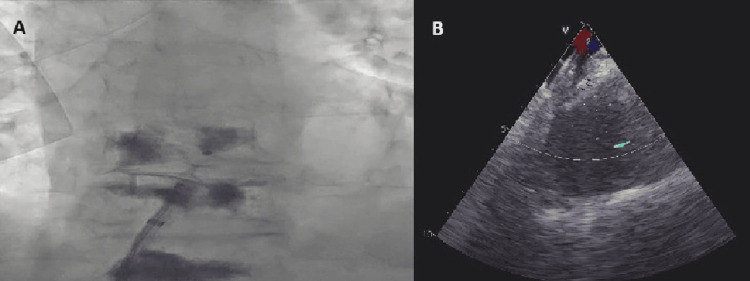
(A) Device deployment showing the two disks of the patent foramen ovale (PFO) occluder across the gap; (B) Intracardiac echo after device deployment demonstrating the device as hyperechoic in the right position.

**Table 2:** Timeline of orthodeoxia and oxygen requirement.

Time Point/Setting	Position	SpO_2_	PaO_2_	O_2_ Requirement
At presentation	Upright	76%	PaO₂ 45 mmHg	15L non-rebreather mask
At presentation	Supine	91%	Not obtained	15L non-rebreather mask
After high-flow nasal cannula (HFNC) supplementation	Upright	82%	Not obtained	100% HFNC
Post-closure	Upright	100%	Not obtained	40% HFNC
At discharge	Upright	93%	Not obtained	Room air

## Discussion

This case report highlights the significance of recognizing POS in patients with positional hypoxia that does not respond to supplemental oxygen. POS has been typically described with ASDs or ventricular septal defects (VSDs) [1]; however, we present a patient with an RLS due to a PFO. She successfully underwent closure with an Amplatzer 30 mm device and had immediate post-operative improvement of her SpO_2_ to 100%.

POS, although rare, is a very diverse syndrome that can have one of three etiologies, namely an intracardiac shunt, an extracardiac shunt, or miscellaneous [2]. From a cardiac standpoint, POS can occur in the context of an ASD, PFO, dilated aortic root, or expanded Chiari network [3-5]. Toffart and colleagues [6] reported 12 patients with concomitant POS-PFO and observed 100% accuracy of TEE for identifying the shunt, and interestingly, right hemidiaphragm elevation in 50% of patients. Similarly, Seward et al. [7] presented seven cases of POS due to intracardiac shunts and recommended diagnosis via tilt-table TEE, in addition to both upright and supine blood gas measurements. Pulmonary etiologies include pneumonectomy, trauma, arteriovenous malformations (AVMs), and hepatopulmonary syndrome [8,9]. Yalonetsky et al. [10] reported a case of PFO closure prior to pneumonectomy to prevent POS, while Welvaart et al. [11] reported three cases of RLS through a PFO due to diaphragmatic relaxation and subsequent right ventricular outflow tract obstruction resulting in POS. Santhirapala et al. [12] reported on 258 patients with pulmonary AVMs, and 29% demonstrated orthodeoxia; interestingly, none endorsed platypnea. Lee et al. reported a case of hepatopulmonary syndrome and POS due to intrapulmonary vascular dilations and shunting [13].

In all etiologies, deoxygenated blood is shunted from the inferior vena cava (IVC) into the systemic system, causing hypoxia [14-16]. Diagnosis requires platypnea, a 4 mmHg decrease in PaO_2_, and an SpO_2_ decrease of >5% when going from supine to upright position [2]. In 1984, POS was demonstrated to occur in a patient with an intracardiac shunt, with neither liver/lung pathology nor elevated right heart pressures [2]. A typical case of POS due to PFO was described by Faller et al. [4], who reported an elderly woman whose SpO_2_ was 89.5% while supine, and 76.6% while upright. Pulmonary function tests and EKG were unremarkable, chest CT demonstrated an aneurysm of the aortic arch and thoracic aorta, and TEE confirmed the finding of a PFO [4].

Intracardiac causes of POS are usually due to a shunt at the interatrial level [2]. Despite the incidence of ASD being less than that of PFO, POS is more commonly seen with the former [5]. A PFO can be imagined as a flap-like tunnel, while an ASD creates a large, fixed, pressure-independent defect. Since the pressure in the left atrium is normally 5-8 mm higher than that of the right atrium, patients with an isolated PFO will not experience POS [2]. A concomitant functional defect that creates transient positional or pressure changes is needed to allow for preferential blood flow through the “tunnel”. Such examples include thoracic kyphoscoliosis, dilation of the ascending aorta, persistent Eustachian valve or Chiari’s network [3].

This case report presents a patient with concomitant aneurysm of the interatrial septum and proximal ascending aorta, increasing the separation of the PFO tunnel and allowing for greater venous return through the RLS while upright [16]. This was demonstrated by Bertaux et al. [17], who described 72 patients with a dilated aortic root on TEE, among whom 19 had a PFO. They determined that the increased aortic size causes the atrial septum to become more mobile. By shortening the length of the septum, the fossa ovalis flap is pulled open, therefore increasing the RLS effect.

Our patient’s history of a known PFO allowed the team to quickly attribute her hypoxia to a cardiac, rather than pulmonary, etiology. Similarly highlighted in other case reports, this patient’s refractory hypoxemia underlines that not all RLS are associated with pulmonary hypertension. This makes it even more important to consider cardiac etiologies as the reason behind a patient’s overt cyanosis [3]. One factor that makes this case unique is not only that her POS occurs in the setting of a PFO rather than ASD, but also that it was accompanied by dilation of the interatrial septum and ascending aorta. Most POS cases demonstrate concomitant functional defects allowing for preferential shunting, as listed above; however, there are a few that characterize POS with an aneurysmal aorta [4]. ASA on its own is rare and is often found in patients with other cardiac defects, particularly ASD and PFO [4]. In patients without pulmonary hypertension, shunting relies on either compression of the right atrium, decreased right ventricular compliance, or distortion of anatomical relations between the vena cava and the left atrium. In both this patient's and Faller et al.’s cases, an enlarged aorta causes rotation of the heart in such a way that caval flow is altered, creating increased RLS [4].

As it is currently recommended to close all PFOs in the setting of POS, given the relatively simple and low-risk procedure, we proceeded with the operation [14]. In the French multicentric study run by Guérin et al. [14], 76 out of 78 patients had successful transcatheter PFO closure, with SpO_2_ increasing from 84.6% to 95.1%. Similar to most patients undergoing PFO closure, she had improvement in both her SpO_2_ (from 79% to 93% at rest) and in her symptoms [2]. She was placed on DAPT for six months afterwards, followed by aspirin indefinitely.

Some limitations to our assessment should be mentioned. Positional shunt severity was not directly quantified before closure, as paired supine and upright PaO_2_ measurements or invasive shunt-fraction measurements were not obtained. However, the diagnosis was supported by the characteristic clinical presentation and improvement in oxygenation after PFO closure. In addition, clear pre- and post-closure TEE was not performed; rather, the assessment was based on transthoracic and intracardiac echocardiograms. Because TEE provides superior visualization of the interatrial septum and closure device, the lack of TEE imaging limited direct anatomic demonstration of the PFO and post-closure device position.

## Conclusions

In conclusion, this case represents an uncommon manifestation of POS due to positional right-to-left shunting across a PFO in the setting of an ASA. The patient was treated with PFO closure, and this led to resolution of symptoms. This case highlights the importance of considering POS in patients with positional, oxygen-refractory hypoxemia, particularly when pulmonary evaluation is unrevealing.
